# Exploring Sleep and Physical Activity among Young Adults Across Asia: A Systematic Literature Review

**DOI:** 10.21315/mjms-06-2025-468

**Published:** 2025-08-30

**Authors:** Vanida Tian, Fatanah Ramlee, Hazalizah Hamzah, Teck Sen Ng

**Affiliations:** Department of Psychology, Faculty of Human Development, Universiti Pendidikan Sultan Idris, Tanjong Malim, Perak, Malaysia

**Keywords:** physical activity, sleep, young adults, health, Asia

## Abstract

Previous studies have examined the association between sleep and physical activity; however, few have systematically reviewed the studies on young adults in Asia. Therefore, the present study aimed to systematically summarise the research on sleep and physical activity among young adults in Asia and critically assess the methodological quality of existing studies. The review process adhered to the Preferred Reporting Items for Systematic Reviews and Meta-Analyses 2020 guidelines, beginning with research question formulation and systematic searches through identification, screening, and eligibility in the Scopus, PubMed, and ScienceDirect databases. Subsequently, the studies underwent quality appraisal, data extraction, and analysis. Thematic analysis organised the findings into three main themes: i) the prevalence of sleep quality and physical activity in Asia; ii) the association between sleep quality and physical activity; and iii) sleep quality and physical activity during the COVID-19 outbreak. These findings reveal a high prevalence of sleep deprivation and poor sleep quality among young adults in Asia, whereas the prevalence of physical activity varies. Moreover, the COVID-19 outbreak negatively affected sleep quality and physical activity. Therefore, proactive measures should be implemented to improve sleep quality and promote physical activity, thereby improving physical and mental health.

## Introduction

Young adults who regularly sleep less than the recommended seven to nine hours per night are at a higher risk of health problems, such as weight gain, obesity, hypertension, diabetes, heart-related diseases, depression, and anxiety (1–4). Lack of sleep can also impair daily executive functions, resulting in longer reaction times and more frequent errors, thereby increasing the risk of accidents. Poor sleep also weakens the immune function and increases susceptibility to infections and illnesses (4, 5). Nevertheless, it is important to acknowledge that individual sleep patterns vary based on genetic, lifestyle, medical, mental, and environmental factors, including screen exposure at bedtime (4, 6, 7).

Regular physical activity is a modifiable factor that improves sleep quality. According to the World Health Organization (WHO), healthy adults aged 18 to 64 years should engage in at least 150 to 300 minutes of moderate-intensity or 75 to 150 minutes of vigorous-intensity aerobic physical activity weekly. When performed sufficiently, physical activity provides significant health benefits, including the prevention of cardiovascular diseases and diabetes and improvement in sleep quality (8, 9). Incorporating muscle-strengthening exercises that involve all major muscle groups on at least two days per week or exceeding either 300 minutes of moderate-intensity or 150 minutes of vigorous-intensity physical activity weekly, is advised (8). However, WHO reported that 31% of adults worldwide do not meet these guidelines (10).

Previous studies demonstrated a bidirectional association between physical activity and sleep in adolescents (11) and adults (12). Physical activity positively correlated with improved sleep quality. Individuals engaged in moderate physical activity had reported better sleep quality, shorter sleep onset, longer sleep duration, and higher sleep maintenance efficiency than sedentary individuals (9, 11). This interaction suggests that improving one’s own behaviour can positively influence others. However, few studies have systematically reviewed these variables among young adults, especially in Asian contexts.

A systematic literature review (SLR) is a replicable, scientific, and transparent method designed to minimise bias by comprehensively gathering and evaluating available studies against predetermined criteria (13). To address the aforementioned research gaps, we formulated the main research question: “What are the prevalence and associations between sleep quality and physical activity among young adults in Asia?” The primary objective of the current study was to systematically summarise existing research on sleep and physical activity among young adults, and to evaluate the methodological quality of the included studies to guide future research and intervention strategies.

## Methods

### Review Protocol – PRISMA 2020

This SLR adhered to the Preferred Reporting Items for Systematic Reviews and Meta-Analyses 2020 (PRISMA 2020) guidelines, because it is comprehensive, and allows readers to assess the methodological rigour, appropriateness, replicability, and applicability of the methods, thereby increasing the trustworthiness of the findings (14). First, research questions were formulated using the PICo method, where “P” stands for Problem or Population, “I” for Interest, and “Co” for Context. Second, document searching began, which involved identification, screening, and eligibility processes, and was followed by quality appraisal based on the criteria adapted from Hong et al. (15). The quality of each selected article was assessed before inclusion. Finally, selected articles underwent data extraction, analysis, and thematic synthesis. Data extraction was driven by the research question, “What are the prevalence and associations between sleep quality and physical activity among young adults in Asia?” The review protocol was registered in PROSPERO, an international prospective register for systematic reviews (reference number: CRD42024555111).

### Formulation of Research Question

The research questions were formulated using the PICo framework to assist the authors in developing suitable review questions. The PICo framework focuses on the Population or Problem, Interest and Context. In this study, the population of interest was young adults with sleep quality and physical activity as the primary areas of interest within the context of Asian countries. This led to the main research question: “What are the prevalence and associations between sleep quality and physical activity among young adults in Asia?”

### Systematic Searching Strategies

**Figure 1:**
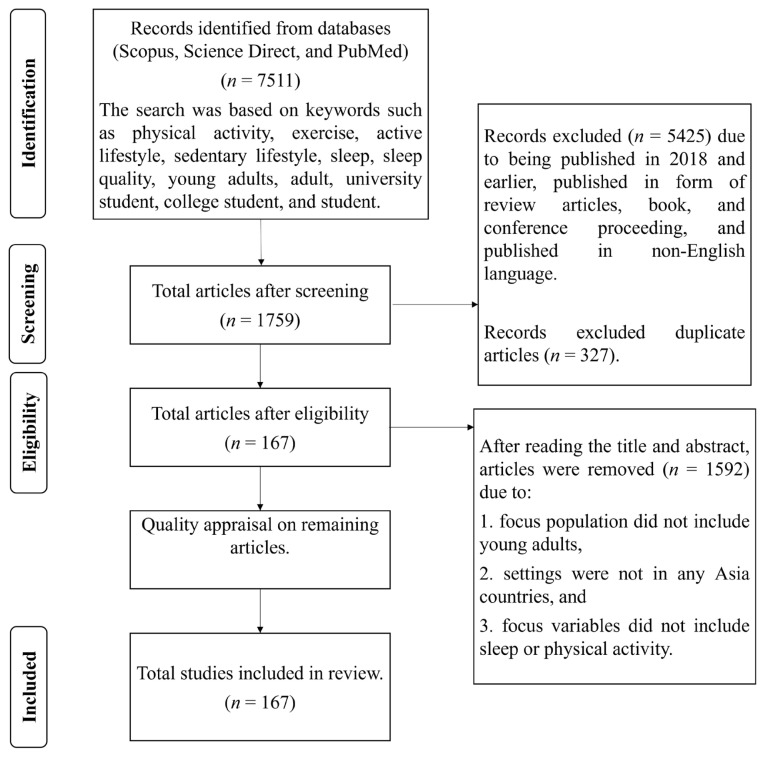
Flowchart of the searching process

The systematic identification, screening, and eligibility process proposed by Mohamed Shaffril et al. (16) were adapted for this study. Figure 1 outlines the search processes. These structured processes allowed for the comprehensive retrieval of relevant articles and facilitated a systematic synthesis.

#### Identification

Based on the formulated research questions, the following four keywords were identified: physical activity, sleep, young adults, and Asia. Synonyms, related terms, and variations were gathered using an online thesaurus, reviewing keywords from previous studies, and consulting expert opinions. Synonyms and related keywords, including exercise, active lifestyle, sedentary lifestyle, sleep quality, adults, university students, college students, and students were added. These keyword combinations were processed using search functions, including field code functions, phrase searching, wildcards, truncation, and Boolean operators, in two databases: Scopus and PubMed (Table 1). Manual searches for these keywords were conducted using the ScienceDirect database. Initially, the keyword “Asia” was not used, as it could limit search results to articles explicitly mentioning “Asia,” potentially excluding relevant studies from Asian countries.

#### Screening

The screening process considered the concept of “research field maturity” (17), limiting included articles to those published between 2019 and 2023. This timeline was selected to ensure the inclusion of sufficient and recent studies in the representative review. Additionally, the review was restricted to empirical research articles that presented primary data and were published in English. Articles from populations other than young adults (i.e., aged 20 to 30 years) were excluded; this age range (20 to 30 years) aligns with the widely accepted definition of young adulthood in the developmental psychology and public health literature (18). This screening process excluded 5,425 articles that did not meet the inclusion criteria, and 327 duplicate articles were removed. Table 2 summarises the inclusion and exclusion criteria of this study.

#### Eligibility

The eligibility phase involved manually examining the articles retrieved after screening to ensure consistency with the research question criteria. The titles and abstracts of the articles were reviewed, resulting in the exclusion of 1,592 articles, as i) the population was not young adults; ii) the settings were outside Asian countries; and iii) the studies did not include sleep or physical activity variables. Ultimately, 167 articles were selected for methodological quality assessment.

### Quality Appraisal

Quality appraisal (QA) was independently conducted by VT (Author 1) and NTS (Author 4) to evaluate the quality of the selected articles (Table S1). The criteria for scoring were yes = 1, partly = 0.5, and no = 0. Mean scores from both authors were calculated. After a critical appraisal, no articles were excluded, leaving 167 articles for review. The QA criteria were as follows:

QA1: Is the purpose of study clearly stated?
QA2: Is the interest and usefulness (significance) of study clearly presented?
QA3: Is the study methodology clearly established?
QA4 Is the concept or approach of study clearly defined?
QA5: Is the work compared or measured with other similar work?
QA6: Are the limitations of study clearly mentioned?

### Data Extraction and Analysis

The data extraction process focused on answering the research question “What are the prevalence and associations between sleep quality and physical activity among young adults in Asia?” Data relevant to this question were extracted from reviewed articles. Thematic analysis identified three themes: i) the prevalence of poor sleep quality and physical activity in Asia; ii) associations between sleep quality and physical activity; and iii) sleep quality and physical activity during the COVID-19 outbreak. Themes were reviewed for accuracy and relevance, and the extracted data were identified, organised, and presented accordingly.

## Results

### Background of Selected Studies

**Figure 2:**
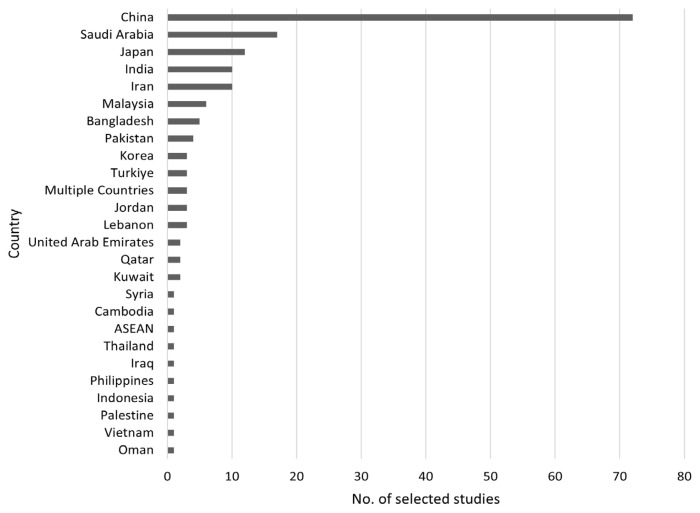
Countries where the selected studies were conducted

**Figure 3:**
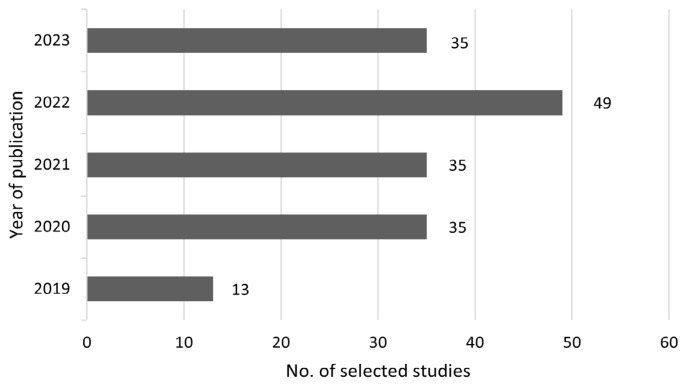
Year of publication of selected studies

Of the 167 selected articles, all included populations were from Asia. Specifically, 72 studies were conducted in China, 17 in Saudi Arabia, 12 in Japan, 10 each in India and Iran, six in Malaysia, five in Bangladesh, and four in Pakistan. Three studies each were conducted in Korea, Türkiye, multiple Asian countries, Jordan, and Lebanon. Two studies each from the United Arab Emirates, Qatar, and Kuwait were reviewed, along with one study each from Syria, Cambodia, ASEAN countries collectively, Thailand, Iraq, the Philippines, Indonesia, Palestine, Vietnam, and Oman (Figure 2). The articles selected were published within the past five years, from 2019 to 2023: 13 articles were published in 2019, 35 each in 2020 and 2021, 49 in 2022, and the most recent 35 articles in 2023 (Figure 3).

### The Developed Themes

The thematic analysis yielded three major themes (Table S2):

Theme 1: Prevalence of sleep quality and physical activity in Asia
Theme 2: Associations between sleep quality and physical activity
Theme 3: Sleep quality and physical activity during COVID-19 outbreak

#### Prevalence of Sleep Quality and Physical Activity in Asia

Of the 167 articles reviewed, 32 reported prevalence data on sleep quality and physical activity among young adults in Asia (Table S2). Sleep deprivation and poor sleep quality are common among young adults in Asia. Studies have indicated that many young adults from countries including Saudi Arabia, Pakistan, Malaysia, Indonesia, Jordan, the United Arab Emirates, Vietnam, Kuwait, China, India, and Japan consistently fail to achieve the recommended seven to nine hours of sleep (4, 19). Poor sleep quality, which is characterised by prolonged sleep latency, sleep disorders, and short sleep duration, is prevalent. Physical activity affects sleep with less active individuals reporting poorer sleep quality (20). Gender-based findings are inconsistent; some studies indicate a higher prevalence of poor sleep among females, while others report the opposite.

All studies comparing physical activity according to gender concluded that males were generally more physically active than women. However, the findings on physical activity levels varied significantly among Asian countries. For instance, Ge et al. (21) reported low physical inactivity among college students, whereas Tao et al. (2) reported contradictory findings. Similarly, studies from Saudi Arabia reported conflicting results; Abdulrahman et al. (22) noted that only 4.3% of medical students exercised daily for 30 minutes or more, whereas Albikawi (23) reported that over 50% of female nursing students engaged regularly in physical activity. A high prevalence of physical inactivity was observed in Malaysia, India, Jordan, and Cambodia, while Pakistan and Kuwait reported a high prevalence of physical activity among young adults.

#### Association between Sleep Quality and Physical Activity

**Figure 4:**
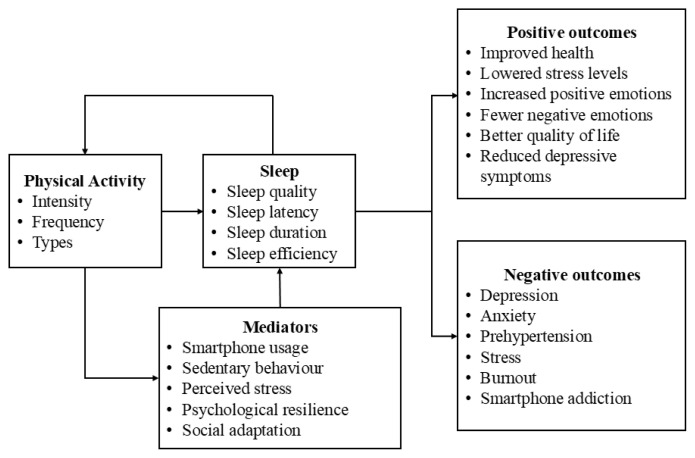
Association between sleep quality and physical activity

Among the 167 articles reviewed, 112 examined the association between sleep and physical activity (Table S2). Figure 4 summarises the association between sleep quality and physical activity. Studies consistently show that individuals with sleep problems are more likely to become physically inactive. Physical inactivity is linked to sleep problems such as poor sleep quality, sleep onset latency exceeding 30 minutes, and sleep deprivation. The risk of poor sleep quality further increases when low physical activity levels are combined with smartphone use or extensive sedentary behaviour. Although many studies have identified a positive association, others have found no significant relationship between sleep and physical activity (24–27).

Regular exercise alleviates academic stress and enhances sleep quality (28). Physically active individuals demonstrate better sleep quality than their inactive peers. Physical activity indirectly influences sleep quality through mediating factors such as mobile phone usage (29), perceived stress (30), psychological resilience, and social adaptation (31). Sleep quality and efficiency also mediated the relationship between physical activity levels and inhibitory control performance (32), indicating that individuals who engage in regular physical activity tend to experience better-regulated sleep patterns, which, in turn, support cognitive processes.

The intensity of physical activity affects sleep differently; higher intensity physical activity combined with reduced sleeping hours reportedly enhances sleep quality (33). Regular aerobic exercise consistently improves sleep quality (34). Under sleep deficient conditions, low-intensity aerobic exercise is preferable to moderate or high-intensity aerobic exercise to reduce stress and maintain hormone levels (35). Moderate-intensity aerobic exercise is also effective in mitigating cognitive impairment caused by sleep deficiency (36, 37).

Sleep and physical activity independently correlated with factors like health status, lifestyle, and psychological issues. Breakfast consumption was associated with better sleep quality and regular physical activity. Frequent breakfast consumers exhibited shorter sleep duration (38), better sleep quality, and higher exercise frequency (39), whereas skipping breakfast was associated with inadequate physical activity and sleep problems (25, 40). Importantly, the relationship between nutrition, sleep, and physical activity suggests the need for an integrated approach to health promotion, particularly among young adults navigating transitional life phases.

High physical activity levels consistently correlated with a range of positive health outcomes, including better sleep quality, longer sleep duration, subjective health improvements, lowered stress levels, reduced depressive symptoms, increased positive emotions, fewer negative emotions, and better quality of life. Conversely, reduced physical activity, particularly when combined with poor sleep quality, was correlated with a cluster of adverse psychological and physiological conditions. These included higher rates of depression, pre-hypertension, stress, burnout, smartphone addiction, and anxiety. Such patterns may reflect a negative behavioural cycle in which physical inactivity exacerbates mental distress, which in turn disrupts sleep and motivation for exercise (41). The bidirectional nature of these associations highlights the importance of separately addressing sleep and physical activity.

#### Sleep Quality and Physical Activity During COVID-19 Outbreak

A total of 47 articles reported sleep quality and physical activity in young adults during the COVID-19 pandemic (Table S2). During this period, strict lockdown measures implemented across countries restricted movement and altered lifestyles, significantly affecting daily routines, including physical activity and sleep. As a result, opportunities for outdoor physical activity diminished, and individuals spent more time indoors. Most studies have reported poor sleep quality, delayed sleep time, irregular sleep schedules, and longer sleep duration. However, some studies have found that individuals maintain good sleep quality or even report improvements during lockdowns (42).

Physical activity mediates the relationship between sleep and mental health, emphasising the importance of maintaining physical activity for improving sleep and psychological well-being (43). Gender differences were also evident, with female students reporting higher incidences of sleep and mental health disorders than their male counterparts (44, 45). Poor sleep quality and shorter sleep duration during the COVID-19 pandemic are linked to anxiety, stress, post-traumatic stress disorder (PTSD) and depression. Al-Musharaf (1) reported that depression is correlated with reduced physical activity and increased sedentary behaviour. Another contributing factor was the increased use of electronic devices as students turned to screening for remote learning, social interaction, and entertainment which negatively impacted sleep quality during the pandemic, leading to prolonged sleep latency and poorer sleep quality. Individuals exposed to COVID-19 also experience significant sleep disturbance, anxiety, and stress (46). These findings indicate the multifactorial nature of sleep and mental health challenges experienced during the pandemic.

Regular physical activity during lockdown was consistently associated with improved sleep quality, reduced negative emotions, and enhanced mental health outcomes (47–50). These findings highlight the critical role of maintaining daily movement even under restrictive situations. Nakahara-Gondoh et al. (51) recommended increasing the number of daily steps by more than 1,000 steps to increase happiness. Physical inactivity and increased sedentary behaviour during the COVID-19 pandemic have been widely reported in China, Iran, Jordan, India, Thailand, Türkiye, Japan, and Saudi Arabia. Surprisingly, despite reduced physical activity and increased sedentary behaviour, some individuals experienced weight loss (52). Rafraf et al. (53) found that Iranian college students prefer household chores to aerobic exercise and spend a significant amount of time sitting during work or leisure activities. However, increased screen time was negatively associated with physical activity levels (7).

## Discussion

### Prevalence and Association between Sleep Quality and Physical Activity among Young Adults in Asia

These findings indicate that young adults across many Asian countries have a high prevalence of sleep deprivation (i.e., regularly sleeping less than the recommended seven to nine hours per night) and poor sleep quality. A recent study among young adults in Malaysia reported a total sleep time of less than six hours, as measured by actigraphy and sleep diaries (54). These results align with the broader international patterns. For example, a national study conducted in the United States reported that one-third of its adult population had a short sleep duration of less than seven hours daily (55). Similarly, the Australian Institute of Health and Welfare reported an estimated prevalence of short sleep between 12% to 18% of Australian adults, with prevalence highest among young adults compared to other age groups (56).

Chronic insufficient sleep and poor sleep quality are associated with health problems, such as weight gain, obesity, hypertension, diabetes, heart-related diseases, depression, and anxiety (1, 3, 57–59). In addition to these long-term consequences, inadequate sleep significantly impairs executive function, leading to slower reaction times, increased errors, weakened immune function, and a higher risk of death (4, 5). Sleep deprivation and poor sleep quality among young adults may result from delayed bedtime due to smartphone use (27) or sleep debt caused by busy or packed daily schedules. Individuals might reduce their sleep duration to compensate for leisure activities, resulting in drowsiness and reduced focus on the subsequent day (60). Young, working adults, particularly those with prolonged working hours or shift work, are more vulnerable to poor sleep hygiene. Irregular work schedules can disrupt normal sleep cycles, while the accompanying behaviour or unhealthy lifestyles, such as cigarette smoking, physical inactivity, or alcohol consumption, further exacerbate sleep (61). These results suggest a complex association between social, occupational, and behavioural factors that influence sleep health in this age group and highlight the importance of tailored public health strategies aimed at improving sleep hygiene and promoting physical activity as a protective factor.

Sleep deprivation is also prevalent among university students, largely because of the demanding nature of their daily schedules. These typically involve balancing academic obligations and responsibilities such as attending classes, participating in sports, engaging in social activities, and completing assignments (62, 63). The cumulative strain on these responsibilities often leads to delayed sleep onset, shortened sleep duration, and reduced sleep quality. Sleep disturbances have been shown to negatively affect cognitive functioning, concentration, and academic performance (64). Moreover, students’ lifestyle factors, such as physical inactivity, prolonged sedentary behaviour, smoking, screen time before bed, academic procrastination and heightened academic stress, further exacerbated poor sleep outcomes (28, 64, 65).

Regular physical activity offers numerous benefits, including improved mental health, emotional well-being, and an enhanced quality of life (2, 66). Several studies have reported an association between physical activity and sleep quality. However, some studies have reported contradictory results showing no significant associations between these variables (24, 27, 67). Young adults who met the recommended physical activity levels reported lower levels of stress, depression, and anxiety (67). However, findings regarding physical activity prevalence among young adults vary considerably, with some studies reporting high physical activity levels and others indicating that young adults do not meet the recommended physical activity levels.

Among students, low physical activity was often due to factors such as sedentary behaviour, lack of time and motivation, social influences, limited sports facilities, and stress (2, 68). Academic commitment often leads students to prioritise studying over other activities, particularly during examination periods, resulting in increased sedentary behaviour and heightened stress. Moreover, university-based sports facilities often operate with limited accessibility, which further limits students’ physical activity options (68).

Social factors significantly influence student engagement in physical activities. Positive social support from family and friends increases motivation for physical activity (2, 68). Gender differences also play a role, with males typically being more active than females. Male students often engage in sports, outdoor activities, socialising, and electronic gaming, whereas female students typically spend more time on household tasks, dancing, studying, internet surfing and shopping (22, 24). These differences in activities may contribute to the variations in physical activity levels according to gender. However, there is no clear consensus regarding the optimal intensity of physical activity (69).

### Sleep and Physical Activity During the COVID-19 Outbreak

During the COVID-19 pandemic, there has been an observed increase in poor sleep quality and altered sleep duration, along with reduced physical activity among young adults across Asia. These disruptions were closely linked to reduced levels of physical activity, largely because of government-imposed lockdowns and widespread movement restrictions aimed at preventing the spread of the virus. Although essential for public health containment, these measures significantly affect physical, emotional, and psychological well-being. Factors such as fear of infection, uncertainty about the future, prolonged confinement, rising domestic violence, and financial challenges contribute to increased anxiety, stress, and depression, which subsequently worsen sleep quality (70, 71). Ironically, although confinement results in increased sleep duration for some individuals owing to limited activity options at home, the quality of sleep often declines owing to disrupted social rhythms, excessive screen exposure, and a sedentary lifestyle.

Lockdown and confinement further reduced physical activity and increased sedentary behaviour. Although the WHO recommends 150 to 300 minutes of moderate-intensity or 75 to 150 minutes of vigorous-intensity aerobic activity per week, lockdown restrictions severely limit outdoor physical activity. Additionally, the closures of sports and recreational facilities have limited physical activity options for confined indoor spaces at home (72). Furthermore, social distancing measures and remote-work policies have encouraged sedentary lifestyles (73).

Home confinement favours sedentary behaviours, including napping, television viewing, and increased social media use, thus reducing the overall energy expenditure (74). Consequently, a weight gain was anticipated. Studies have also reported increased consumption of unhealthy foods such as meat, sweets, snacks, and sugary beverages, alongside skipping breakfast during the lockdown (72, 74, 75). However, some individuals experienced no weight change or eventual weight loss, likely due to reduced fast food consumption, increased home-cooked meals (52), greater availability of free time for exercise, and increased physical activity to mitigate the stress and negative emotions associated with the pandemic (57). Thus, the impact of confinement varies according to the individual’s efforts, financial situation, and overall physical and psychological health.

During the COVID-19 pandemic, the downsides of confinement were observed, specifically increased poor sleep quality and physical inactivity. Therefore, preventive measures against future health crises must be considered. One suggestion is to introduce weight management guidelines which clearly state suitable physical activities that can be conducted in accordance with the situation (52). Promoting regular daytime naps can also effectively improve sleep quality and reduce psychological stress (76). As the pandemic affects mental health, which subsequently affects sleep patterns and physical activity levels, psychological support is of utmost importance. Social media and online consultations by certified professionals are a few platforms that could promote mental health awareness and provide psychological aid and care during crises (49, 52).

## Conclusion

This study systematically reviewed articles from three major databases to examine the prevalence and relationships between sleep and physical activity among young adults in Asia. Each included study was critically appraised for methodological quality, and thematic analysis was employed to organise the findings into three core themes: i) the prevalence of poor sleep quality and physical activity in Asia; ii) the association between sleep quality and physical activity; and iii) sleep quality and physical activity during the COVID-19 outbreak. These findings revealed a consistently high prevalence of sleep deprivation and poor sleep quality among young Asian adults. However, the prevalence of physical activity varies considerably across studies. Factors such as academic stress, screen exposure before bed, and lifestyle shifts were frequently implicated. During the COVID-19 outbreak, home confinement further exacerbated poor sleep outcomes, altered sleep duration, and decreased physical activity, emphasising the compounding effects of societal disruptions on young adults’ health behaviours.

Despite the valuable insights provided by the present SLR, some limitations warrant consideration. First, this review included only three databases, namely, Scopus, PubMed, and ScienceDirect, owing to access restrictions, which may have excluded relevant studies. Future studies should explore additional databases to ensure a comprehensive coverage. Second, the review included only English-language articles published in Asia, potentially overlooking culturally diverse studies published in regional languages. This language restriction may have limited the diversity of the perspectives and should be addressed in future research. Finally, the study did not include a meta-analysis synthesis, nor did it critically investigate the specific measurement tools for sleep and physical activity, such as actigraphy and pedometers, which could potentially affect the accuracy of the results. Nevertheless, this review highlights the urgent need for proactive measures to improve sleep quality and promote physical activity among young adults in Asia to enhance their physical and mental health. It is important to consider hybrid intervention programmes that simultaneously target both behaviours, particularly in high-stress environments during periods of social disruption. Improving sleep and physical activity in this population is important for improving their physical health, cognitive function, emotional well-being, and long-term resilience.

## Supplementary Materials

**Table S1:** The criteria used to determine the rigour of the methodology and analysis used in the selected articles

No.	Study	QA1	QA2	QA3	QA4	QA5	QA6	Total score	Inclusion in the review
1	Abdulla et al. (2023)	1	1	1	1	1	1	6	Yes
2	Abdulrahman et al. (2021)	1	0.75	1	0.5	1	1	5.25	Yes
3	Adachi et al. (2022)	1	0	1	0.5	0	1	3.5	Yes
4	Ai et al. (2021)	1	1	1	1	1	1	6	Yes
5	Albikawi (2023)	1	0.5	1	1	1	1	5.5	Yes
6	Albqoor and Shaheen (2021)	1	0.75	1	1	1	1	5.75	Yes
7	Albutaysh et al. (2020)	1	1	1	1	0	1	5	Yes
8	Aldhwayan and Alabdulkader (2022)	1	0	1	0.5	1	1	4.5	Yes
9	Al-Houqani et al. (2020)	1	0	1	1	1	0.5	4.5	Yes
10	Alkatan et al. (2021)	1	0.75	1	0.5	1	1	5.25	Yes
11	Al-Musharaf (2020)	1	0.5	1	0.5	0.75	1	4.75	Yes
12	Al-Musharaf (2022)	1	0.25	1	0.75	1	1	5	Yes
13	Al-Musharaf et al. (2021)	1	0.75	1	1	0.75	1	5.5	Yes
14	Alotaibi et al. (2023)	1	0.5	0.5	0	0.5	1	3.5	Yes
15	Alotaibi et al. (2022)	1	1	1	0.5	1	0.5	5	Yes
16	Al-Sayegh et al. (2020)	1	0.75	1	0.75	1	1	5.5	Yes
17	Alshammari et al. (2022)	1	0	1	1	1	0.5	4.5	Yes
18	Alshehri et al. (2023)	1	1	1	1	1	1	6	Yes
19	Alsulami et al. (2023)	1	0	1	0.5	1	1	4.5	Yes
20	Alzamil et al. (2019)	1	0.5	1	0.5	1	0.5	4.5	Yes
21	Amzajerdi et al. (2023)	1	0	1	0.5	1	0	3.5	Yes
22	Balhareth et al. (2021)	1	1	1	0.25	0.5	1	4.75	Yes
23	Bazyar et al. (2020)	1	1	1	1	1	1	6	Yes
24	Boozari et al. (2022)	1	0.5	1	1	1	0.5	5	Yes
25	Bu et al. (2021)	1	1	1	1	1	1	6	Yes
26	Cahuas et al. (2019)	1	0.5	1	1	1	1	5.5	Yes
27	Chaabna et al. (2022)	1	0.25	1	1	0.75	0	4	Yes
28	D. P. Chao (2023)	0.5	1	0.5	1	1	1	5	Yes
29	C. Chao et al. (2022)	1	0	1	1	1	0.5	4.5	Yes
30	L. Chao et al. (2022)	1	0.5	1	1	1	1	5.5	Yes
31	Cheema et al. (2022)	1	1	0.5	0	0	0.5	3	Yes
32	M. Chen et al. (2022)	1	0.5	1	1	1	1	5.5	Yes
33	Q. Chen et al. (2022)	1	0.5	1	1	1	1	5.5	Yes
34	Z. Y. Chen et al. (2021)	1	0	1	0	1	1	4	Yes
35	H. Chen et al. (2022)	1	0.5	1	0.75	0.75	1	5	Yes
36	Chun et al. (2021)	1	1	1	1	1	1	6	Yes
37	Dai et al. (2021)	1	0.5	1	1	0.5	1	5	Yes
38	Din et al. (2019)	1	0	1	0.5	0.5	0.5	3.5	Yes
39	Eyupoglu et al. (2022)	1	0	1	0	1	1	4	Yes
40	Ezati et al. (2020)	1	0	1	1	1	0	4	Yes
41	Feng et al. (2022a)	1	0.5	1	0.5	1	1	5	Yes
42	Feng et al. (2022b)	1	0	1	1	1	1	5	Yes
43	Gao et al. (2023)	1	1	1	0.5	0.5	1	5	Yes
44	Ge et al. (2019)	0.25	0.75	1	0.5	0.5	1	4	Yes
45	Ghrouz et al. (2019)	1	0.5	1	1	1	0	4.5	Yes
46	Ghrouz et al. (2021)	1	0	1	1	0.75	1	4.75	Yes
47	Güneşer and Hİm (2022)	1	1	1	1	1	0	5	Yes
48	Guo et al. (2020)	1	0	1	1	1	1	5	Yes
49	Haidar et al. (2019)	1	1	1	1	1	0.5	5.5	Yes
50	Halat et al. (2023)	1	1	1	1	1	0.5	5.5	Yes
51	Hammoudi et al. (2021)	1	0.5	1	1	1	1	5.5	Yes
52	Hao et al. (2023)	1	0	1	1	1	0.5	4.5	Yes
53	Hasan and Moustafa (2022)	1	0	1	0	1	0	3	Yes
54	Hashimoto et al. (2021)	1	0	0.5	1	0	0.5	3	Yes
55	Hosen et al. (2021)	1	0.5	1	1	1	1	5.5	Yes
56	Hsu and Chiang (2020)	1	0	1	0.5	1	0.5	4	Yes
57	Iqbal et al. (2021)	1	1	1	0.5	0.5	1	5	Yes
58	Islam et al. (2020)	1	1	1	1	1	1	6	Yes
59	Y. Ji et al. (2022)	1	0	1	1	1	1	5	Yes
60	C. Ji et al. (2022)	1	0	1	1	1	0.5	4.5	Yes
61	Jiang et al. (2020)	1	0	1	0	1	1	4	Yes
62	Kailani et al. (2023)	1	1	0.5	1	0.5	0	4	Yes
63	Kalal et al. (2023)	1	1	1	1	0	1	5	Yes
64	Kalpana et al. (2022)	1	0	1	1	1	0.5	4.5	Yes
65	Karimy et al. (2020)	1	0.5	1	0.5	1	1	5	Yes
66	Karimy et al. (2019)	1	0.5	0.5	1	1	0.5	4.5	Yes
67	Kathem et al. (2021)	1	0	1	1	1	1	5	Yes
68	Khraiwesh et al. (2023)	1	0	1	1	1	0.5	4.5	Yes
69	Kojima et al. (2020)	1	0	1	1	1	1	5	Yes
70	Kolhar et al. (2021)	1	0	0.5	0	0.5	0	2	Yes
71	Kundu et al. (2021)	1	0	1	1	1	1	5	Yes
72	Kwok et al. (2021)	1	0	1	1	1	1	5	Yes
73	Lee et al. (2022)	1	0	1	1	1	0	4	Yes
74	Lee et al. (2020)	1	1	1	1	0.75	1	5.75	Yes
75	Li and Guo (2023)	1	0.5	1	1	1	1	5.5	Yes
76	Li and Li (2022)	1	0.75	1	1	0.5	1	5.25	Yes
77	Li et al. (2022)	1	0.5	1	1	1	1	5.5	Yes
78	Li et al. (2021)	1	0	1	1	1	1	5	Yes
79	Liang et al. (2021)	1	1	1	1	1	1	6	Yes
80	Lin and Liu (2023)	1	0	1	0	1	0.5	3.5	Yes
81	Lin et al. (2019)	1	0	1	1	1	1	5	Yes
82	Liu and Zhang (2022)	1	0	1	1	1	0	4	Yes
83	Liu et al. (2023)	1	0.5	1	1	1	0.5	5	Yes
84	Liu et al. (2021)	1	1	1	1	1	1	6	Yes
85	Liu et al. (2021)	1	0	1	1	0	0.5	3.5	Yes
86	Lu et al. (2022)	1	0.5	1	1	1	0.5	5	Yes
87	Lu et al. (2021)	1	0.25	1	0.25	0.5	1	4	Yes
88	Luo et al. (2023)	1	0	1	1	1	0.5	4.5	Yes
89	Ma et al. (2020)	1	0	1	1	1	1	5	Yes
90	Mahfouz et al. (2020)	1	0	1	1	1	1	5	Yes
91	Malik et al. (2019)	0.5	1	0.75	0.75	0.75	1	4.75	Yes
92	Mansouri et al. (2020)	0.5	0	1	0	1	1	3.5	Yes
93	Masaad et al. (2021)	1	0	1	1	1	1	5	Yes
94	Mato and Tsukasaki (2020)	1	0.25	1	0.5	1	1	4.75	Yes
95	Matsunaga et al. (2021)	1	0	1	1	1	1	5	Yes
96	Mishra et al. (2022)	1	0	1	0.5	1	0.5	4	Yes
97	Naito et al. (2021)	1	0.5	1	0.75	0.75	1	5	Yes
98	Nakahara-gondoh et al. (2022)	1	0	1	0.5	0.5	0.5	3.5	Yes
99	Nazzal and Berte (2020)	1	0.5	1	0.5	1	1	5	Yes
100	Omar and Kudin (2023)	1	0	1	1	1	1	5	Yes
101	Park et al. (2022)	1	0.5	1	1	1	1	5.5	Yes
102	Park et al. (2021)	1	0	1	1	1	0.5	4.5	Yes
103	Parkash et al. (2023)	1	0	1	1	1	1	5	Yes
104	Peltzer and Pengpid (2019)	1	0	1	0.5	0.5	0.5	3.5	Yes
105	Pengpid and Peltzer (2019)	1	0.5	1	1	1	0.5	5	Yes
106	Pengpid and Peltzer (2020)	1	0.25	1	0.5	1	1	4.75	Yes
107	Pengpid and Peltzer (2020)	1	0.5	1	1	1	1	5.5	Yes
108	Pham et al. (2021)	1	0	1	1	1	1	5	Yes
109	Prabhat et al. (2022)	1	0	1	0.5	1	0.5	4	Yes
110	Qaiser et al. (2020)	0.5	0	1	0.5	0.75	0.75	3.5	Yes
111	Qiu et al. (2022)	1	0.5	1	1	1	1	5.5	Yes
112	Rafraf et al. (2023)	1	0.5	1	0.5	1	0.5	4.5	Yes
113	Rajasekhar et al. (2023)	1	0.5	1	1	1	1	5.5	Yes
114	Ren et al. (2021)	1	0.5	1	1	1	1	5.5	Yes
115	Saat et al. (2021)	1	0	1	1	1	1	5	Yes
116	Saeed and Javed (2021)	1	0	1	0	1	0	3	Yes
117	Sahasakul et al. (2023)	1	0	1	1	1	1	5	Yes
118	Sharafi et al. (2020)	1	0	1	1	1	0.5	4.5	Yes
119	Shi et al. (2023)	1	0	1	0.5	1	0.5	4	Yes
120	Shimamoto et al. (2023)	1	0	1	1	1	1	5	Yes
121	Shimamoto et al. (2021)	1	0	1	1	1	1	5	Yes
122	Singla et al. (2023)	1	0.5	1	0	1	0.5	4	Yes
123	Sofyana et al. (2022)	1	0	1	1	1	1	5	Yes
124	Sok et al. (2020)	1	0	1	1	1	1	5	Yes
125	Soltani et al. (2022)	1	0	1	1	1	0.5	4.5	Yes
126	Srinivasan et al. (2021)	1	0.5	1	1	1	0	4.5	Yes
127	Su et al. (2022)	1	0.5	1	1	1	0.5	5	Yes
128	Swed et al. (2023)	0.5	0.5	1	1	1	1	5	Yes
129	Tao et al. (2019)	1	0.75	0.75	0.75	1	1	5.25	Yes
130	Tasnim et al. (2020)	1	0.5	1	1	1	1	5.5	Yes
131	Tomishima et al. (2022)	1	0.5	1	1	1	1	5.5	Yes
132	Uddin et al. (2019)	1	0	1	1	1	0	4	Yes
133	Upadhyay et al. (2023)	1	0	0	0	1	0	2	Yes
134	Villarino et al. (2022)	1	0	1	0	1	0.5	3.5	Yes
135	Wang et al. (2023)	1	0	1	1	1	1	5	Yes
136	Wang et al. (2020)	1	1	1	1	1	1	6	Yes
137	Wang et al. (2020)	1	0.5	1	0.5	0.5	1	4.5	Yes
138	Wang et al. (2020)	1	1	1	1	0	1	5	Yes
139	Wang et al. (2022)	1	0.25	1	0.25	0.75	1	4.25	Yes
140	Watanabe et al. (2022)	1	0	1	1	1	1	5	Yes
141	Wu et al. (2022)	1	0.5	1	1	1	1	5.5	Yes
142	Xiao et al. (2022)	1	1	1	0.5	1	1	5.5	Yes
143	Xu et al. (2023)	1	1	1	0.5	0.5	1	5	Yes
144	Yang et al. (2023)	1	0.5	1	1	1	1	5.5	Yes
145	Yang et al. (2022)	1	1	1	1	1	1	6	Yes
146	Yang et al. (2020)	1	1	1	0.5	0.25	0.75	4.5	Yes
147	Yang et al. (2020)	1	0.5	1	1	0.5	1	5	Yes
148	Ye et al. (2022)	1	1	1	1	1	1	6	Yes
149	Yin et al. (2022)	1	0	1	0.5	1	0.5	4	Yes
150	Yoshimura et al. (2020)	1	0	1	1	0.5	1	4.5	Yes
151	You et al. (2023)	1	0.5	1	0.5	1	1	5	Yes
152	Yu et al. (2023)	1	1	1	1	1	1	6	Yes
153	Yu et al. (2020)	1	0.5	1	1	1	1	5.5	Yes
154	Yuan et al. (2022)	0.75	0.5	1	1	0.75	0	4	Yes
155	Yücel and Yücel (2022)	1	0	0.5	0.5	1	1	4	Yes
156	Zhai et al. (2020)	1	0.5	1	1	1	1	5.5	Yes
157	Zhai et al. (2021)	1	0	1	1	1	0.5	4.5	Yes
158	Zhang et al. (2023)	1	0.5	1	1	1	1	5.5	Yes
159	Zhang et al. (2023)	0.5	0	1	0.5	1	1	4	Yes
160	Zhang et al. (2022)	1	0	1	1	1	0.5	4.5	Yes
161	Zhang et al. (2022)	1	1	1	0.5	0.5	1	5	Yes
162	Zhang et al. (2020)	1	0.75	1	1	0.5	1	5.25	Yes
163	Zhang, et al. (2022)	1	0	1	1	1	1	5	Yes
164	Zhang et al. (2020)	1	1	1	1	1	1	6	Yes
165	Zhang et al. (2023)	1	0	1	1	1	1	5	Yes
166	Zhao et al. (2022)	1	1	1	1	1	1	6	Yes
167	Zheng et al. (2020)	1	1	1	0	1	1	5	Yes

**Table S2:** Findings

No.	Study	Country	Year of publication	Theme 1	Theme 2	Theme 3
1	Abdulla et al.	United Arab Emirates	2023		/	
2	Abdulrahman et al.	Saudi Arabia	2021	/		
3	Adachi et al.	Japan	2022		/	
4	Ai et al.	China	2021		/	
5	Albikawi	Saudi Arabia	2023	/		
6	Albqoor and Shaheen	Jordan	2021	/	/	
7	Albutaysh et al.	Saudi Arabia	2020		/	
8	Aldhwayan and Alabdulkader	Saudi Arabia	2022			/
9	Al-Houqani et al.	Oman	2020		/	
10	Alkatan et al.	Kuwait	2021	/	/	
11	Al-Musharaf	Saudi Arabia	2020		/	/
12	Al-Musharaf	Saudi Arabia	2022	/		
13	Al-Musharaf et al.	Saudi Arabia	2021		/	/
14	Alotaibi et al.	Saudi Arabia	2023		/	
15	Alotaibi et al.	Saudi Arabia	2022		/	
16	Al-Sayegh et al.	Kuwait	2020	/		
17	Alshammari et al.	Saudi Arabia	2022			/
18	Alshehri et al.	Saudi Arabia	2023		/	
19	Alsulami et al.	Saudi Arabia	2023	/	/	
20	Alzamil et al.	Saudi Arabia	2019	/		
21	Amzajerdi et al.	Iran	2023		/	
22	Balhareth et al.	Saudi Arabia	2021		/	
23	Bazyar et al.	Iran	2020		/	
24	Boozari et al.	Iran	2022		/	
25	Bu et al.	China	2021		/	
26	Cahuas et al.	China	2019		/	
27	Chaabna et al.	Qatar	2022	/		
28	Chao	China	2023		/	
29	Chao et al.	China	2022		/	
30	Chao et al.	China	2022		/	
31	Cheema et al.	Qatar	2022		/	
32	Chen et al.	China	2022		/	
33	Chen et al.	China	2022			/
34	Chen et al.	Malaysia	2021		/	
35	Chen et al.	China	2022		/	
36	Chun et al.	Korea	2021		/	
37	Dai et al.	China	2021			/
38	Din et al.	Pakistan	2019		/	
39	Eyupoglu et al.	Turkiye	2022			/
40	Ezati et al.	Iran	2020		/	
41	Feng et al.	China	2022a			/
42	Feng et al.	China	2022b			/
43	Gao et al.	China	2023		/	
44	Ge et al.	China	2019	/	/	
45	Ghrouz et al.	India	2019	/	/	
46	Ghrouz et al.	India	2021	/	/	
47	Güneşer and Hİm	Turkiye	2022			/
48	Guo et al.	China	2020		/	
49	Haidar et al.	Lebanon	2019		/	
50	Halat et al.	Lebanon	2023		/	
51	Hammoudi et al.	Lebanon	2021			/
52	Hao et al.	China	2023		/	
53	Hasan and Moustafa	Malaysia	2022			/
54	Hashimoto et al.	Japan	2021			/
55	Hosen et al.	Bangladesh	2021			/
56	Hsu and Chiang	China	2020		/	
57	Iqbal et al.	Saudi Arabia	2021			/
58	Islam et al.	Bangladesh	2020		/	
59	Ji et al.	China	2022			/
60	Ji et al.	China	2022		/	
61	Jiang et al.	China	2020		/	
62	Kailani et al.	Jordan	2023	/	/	
63	Kalal et al.	India	2023			/
64	Kalpana et al.	India	2022	/	/	
65	Karimy et al.	Iran	2020		/	
66	Karimy et al.	Iran	2019		/	
67	Kathem et al.	Iraq	2021		/	
68	Khraiwesh et al.	Jordan	2023			/
69	Kojima et al.	Japan	2020		/	
70	Kolhar et al.	Saudi Arabia	2021		/	
71	Kundu et al.	Bangladesh	2021		/	
72	Kwok et al.	China	2021		/	
73	Lee et al.	Korea	2022			/
74	Lee et al.	China	2020		/	
75	Li and Guo	China	2023		/	
76	Li and Li	China	2022		/	
77	Li et al.	China	2022		/	
78	Li et al.	China	2021		/	
79	Liang et al.	China	2021			/
80	Lin and Liu	China	2023		/	
81	Lin et al.	China	2019		/	
82	Liu and Zhang	China	2022		/	
83	Liu et al.	China	2023			/
84	Liu et al.	China	2021	/	/	
85	Liu et al.	China	2021		/	
86	Lu et al.	China	2022		/	
87	Lu et al.	China	2021	/	/	
88	Luo et al.	China	2023		/	
89	Ma et al.	China	2020	/	/	
90	Mahfouz et al.	Saudi Arabia	2020		/	
91	Malik et al.	Pakistan	2019	/	/	
92	Mansouri et al.	Iran	2020		/	
93	Masaad et al.	United Arab Emirates	2021	/		
94	Mato and Tsukasaki	Japan	2020	/	/	
95	Matsunaga et al.	Japan	2021	/	/	
96	Mishra et al.	India	2022			/
97	Naito et al.	Malaysia	2021	/		
98	Nakahara-gondoh et al.	Japan	2022			/
99	Nazzal and Berte	Palestine	2020		/	
100	Omar and Kudin	Malaysia	2023			/
101	Park et al.	Korea	2022		/	
102	Park et al.	Japan	2021		/	
103	Parkash et al.	Pakistan	2023		/	
104	Peltzer and Pengpid	ASEAN	2019		/	
105	Pengpid and Peltzer	Multiple countries	2019		/	
106	Pengpid and Peltzer	Multiple countries	2020a		/	
107	Pengpid and Peltzer	Multiple countries	2020b		/	
108	Pham et al.	Vietnam	2021	/	/	
109	Prabhat et al.	India	2022			/
110	Qaiser et al.	Malaysia	2020		/	
111	Qiu et al.	China	2022		/	
112	Rafraf et al.	Iran	2023			/
113	Rajasekhar et al.	India	2023		/	
114	Ren et al.	China	2021			/
115	Saat et al.	Malaysia	2021	/		
116	Saeed and Javed	Pakistan	2021			/
117	Sahasakul et al.	Thailand	2023			/
118	Sharafi et al.	Iran	2020		/	
119	Shi et al.	China	2023		/	
120	Shimamoto et al.	Japan	2023			/
121	Shimamoto et al.	Japan	2021		/	
122	Singla et al.	India	2023			/
123	Sofyana et al.	Indonesia	2022	/		
124	Sok et al.	Cambodia	2020	/		
125	Soltani et al.	Iran	2022			/
126	Srinivasan et al.	India	2021		/	
127	Su et al.	China	2022			/
128	Swed et al.	Syria	2023		/	
129	Tao et al.	China	2019	/	/	
130	Tasnim et al.	Bangladesh	2020			/
131	Tomishima et al.	Japan	2022		/	
132	Uddin et al.	Bangladesh	2019		/	
133	Upadhyay et al.	India	2023	/		
134	Villarino et al.	Philippines	2022		/	
135	Wang et al.	China	2023		/	
136	Wang et al.	China	2020		/	
137	Wang et al.	China	2020		/	
138	Wang et al.	China	2020			/
139	Wang et al.	China	2022		/	/
140	Watanabe et al.	Japan	2022			/
141	Wu et al.	China	2022		/	
142	Xiao et al.	China	2022			/
143	Xu et al.	China	2023	/	/	
144	Yang et al.	China	2023		/	
145	Yang et al.	China	2022		/	
146	Yang et al.	China	2020			/
147	Yang et al.	China	2020		/	
148	Ye et al.	China	2022	/	/	
149	Yin et al.	China	2022			/
150	Yoshimura et al.	Japan	2020	/		
151	You et al.	China	2023	/	/	
152	Yu et al.	China	2023		/	
153	Yu et al.	China	2020		/	
154	Yuan et al.	China	2022		/	/
155	Yücel and Yücel	Turkiye	2022			/
156	Zhai et al.	China	2020		/	
157	Zhai et al.	China	2021		/	
158	Zhang et al.	China	2023		/	
159	Zhang et al.	China	2023		/	
160	Zhang et al.	China	2022		/	
161	Zhang et al.	China	2022			/
162	Zhang et al.	China	2020		/	/
163	Zhang et al.	China	2022			/
164	Zhang et al.	China	2020		/	
165	Zhang et al.	China	2023			/
166	Zhao et al.	China	2022		/	
167	Zheng et al.	China	2020			/

## Figures and Tables

**Table 1:** Search string used in selected databases

Database	String
Scopus	TITLE-ABS-KEY ( ( “physical activit*” OR “exercise*” OR “active lifestyle*” OR “sedentary lifestyle*” ) AND ( “sleep” OR “sleep quality” ) AND ( “young adult*” OR “university student*” OR “college student*” ) )
PubMed	(“physical activity” OR “exercise” OR “active lifestyle” OR “sedentary lifestyle”) AND (“sleep” OR “sleep quality”) AND (“young adult” OR “university student” OR “college student”)

**Table 2:** Inclusion and exclusion criteria

Criterion	Inclusion	Exclusion
Timeline	2019 to 2023	2018 and earlier
Population sample	Young adults	Population other than young adults
Setting	Countries in Asia continent	No Asia countries included
Document type	Research articles	Review articles, book, conference proceedings, etc.
Language	English	Non-English

## References

[1] Al-Musharaf S (2020). Prevalence and predictors of emotional eating among healthy young Saudi women during the COVID-19 pandemic. Nutrients.

[2] Tao K, Liu W, Xiong S, Ken L, Zeng N, Peng Q (2019). Associations between self-determined motivation, accelerometer-determined physical activity, and quality of life in Chinese college students. Int J Environ Res Public Health.

[3] Nurismadiana I, Lee K (2018). Factors associated with sleep quality among undergraduate students at a Malaysian public university. Int J Public Health Clin Sci.

[4] Watson NF, Badr MS, Belenky G, Bliwise DL, Buxton OM, Buysse D (2015). Recommended amount of sleep for a healthy adult: a joint consensus statement of the American Academy of Sleep Medicine and Sleep Research Society. J Clin Sleep Med.

[5] Skurvydas A, Zlibinaite L, Solianik R, Brazaitis M, Valanciene D, Baranauskiene N (2020). One night of sleep deprivation impairs executive function but does not affect psychomotor or motor performance. Biol Sport.

[6] Wang F, Bíró É (2021). Determinants of sleep quality in college students: a literature review. Explore.

[7] Chen Q, Dai W, Li G, Ma N (2022). The impact of screen time changes on anxiety during the COVID-19 pandemic: sleep and physical activity as mediators. Sleep Biol Rhythms.

[8] World Health Organization (2020). WHO guidelines on physical activity and sedentary behavior.

[9] Park H, Suh B (2020). Association between sleep quality and physical activity according to gender and shift work. J Sleep Res.

[10] World Health Organization (2024). Physical activity. [Internet].

[11] Master L, Nye RT, Lee S, Nahmod NG, Mariani S, Hale L (2019). Bidirectional, daily temporal associations between sleep and physical activity in adolescents. Sci Rep.

[12] Seol J, Lee J, Park I, Tokuyama K, Fukusumi S, Kokubo T (2022). Bidirectional associations between physical activity and sleep in older adults: a multilevel analysis using polysomnography. Sci Rep.

[13] Linnenluecke MK, Marrone M, Singh AK (2020). Conducting systematic literature reviews and bibliometric analyses. Aust J Manag.

[14] Page MJ, McKenzie JE, Bossuyt PM, Boutron I, Hoffmann TC, Mulrow CD (2021). The PRISMA 2020 statement: an updated guideline for reporting systematic reviews. BMJ.

[15] Hong QN, Pluye P, Fabregues S, Bartlett G, Boardman F, Cargo M (2018). Mixed methods appraisal tool (MMAT), version 2018.

[16] Mohamed Shaffril HA, Samsuddin SF, Abu Samah A (2021). The ABC of systematic literature review: The basic methodological guidance for beginners. Qual Quant.

[17] Kraus S, Breier M, Dasí-Rodríguez S (2020). The art of crafting a systematic literature review in entrepreneurship research. Int Entrep Manag J.

[18] American Psychological Association Adulthood. [Internet].

[19] Hirshkowitz M, Whiton K, Albert SM, Alessi C, Bruni O, DonCarlos L (2015). National Sleep Foundation’s sleep time duration recommendations: methodology and results summary. Sleep Health.

[20] Ghrouz AK, Noohu MM, Manzar D, Bekele BB, Pandi-Perumal SR, Bahammam AS (2021). Short-term insomnia symptoms are associated with level and not type of physical activity in a sample of Indian college students. J Prev Med Hyg.

[21] Ge Y, Xin S, Luan D, Zou Z, Liu M, Bai X (2019). Association of physical activity, sedentary time, and sleep duration on the health-related quality of life of college students in Northeast China. Health Qual Life Outcomes.

[22] Abdulrahman KA, Khalaf AM, Abbas FB, Alanezi OT (2021). The lifestyle of Saudi medical students. Int J Environ Res Public Health.

[23] Albikawi ZF (2023). Perceived stress, physical activity, and insomnia of female nursing university students in Saudi Arabia: a cross-sectional study. Univ J Public Health.

[24] Alkatan M, Alsharji K, Akbar A, Alshareefi A, Alkhalaf S, Alabduljader K (2021). Physical activity and sedentary behaviors among active college students in Kuwait relative to gender status. Kuwait Med J.

[25] Pengpid S, Peltzer K (2020). Skipping breakfast and its association with health risk behaviour and mental health among university students in 28 countries. Diabetes Metab Syndr Obes.

[26] Park I, Díaz J, Matsumoto S, Iwayama K, Nabekura Y, Ogata H (2021). Exercise improves the quality of slow wave sleep by increasing slow wave stability. Sci Rep.

[27] Chen H, Zhang G, Wang Z, Feng S, Li H (2022). The associations between daytime physical activity, while-in-bed smartphone use, sleep delay, and sleep quality: a 24-h investigation among Chinese college students. Int J Environ Res Public Health.

[28] Yuan MZ, Chen CC, Chen IS, Yang CC, Hsu CH (2022). Research on the impact of regular exercise behavior of college students on academic stress and sleep quality during the COVID-19 pandemic. Healthcare.

[29] Xu CY, Zhu KT, Ruan XY, Zhu XY, Zhang YS, Tong WX (2023). Effect of physical exercise on sleep quality in college students: mediating role of smartphone use. PLoS One.

[30] Zhai X, Wu N, Koriyama S, Wang C, Shi M, Huang T (2021). Mediating effect of perceived stress on the association between physical activity and sleep quality among Chinese college students. Int J Environ Res Public Health.

[31] Li Y, Guo K (2023). Research on the relationship between physical activity, sleep quality, psychological resilience, and social adaptation among Chinese college students: a cross-sectional study. Front Psychol.

[32] Li L, Yu Q, Zhao W, Herold F, Cheval B, Kong Z (2021). Physical activity and inhibitory control: The mediating role of sleep quality and sleep efficiency. Brain Sci.

[33] Lu Y, Wiltshire HD, Baker JS, Wang Q (2022). Effects of low-volume high-intensity interval exercise on 24 h movement behaviors in inactive female university students. Int J Environ Res Public Health.

[34] Ezati M, Keshavarz M, Barandouzi ZA, Montazeri A (2020). The effect of regular aerobic exercise on sleep quality and fatigue among female student dormitory residents. BMC Sports Sci Med Rehabil.

[35] Park JS, Kim YJ, Heo W, Kim S (2022). The study of variation of metabolites by sleep deficiency, and intervention possibility of aerobic exercise. Int J Environ Res Public Health.

[36] Kojima S, Abe T, Morishita S, Inagaki Y, Qin W, Hotta K (2019). Acute moderate-intensity exercise improves 24-h sleep deprivation-induced cognitive decline and cerebral oxygenation: a near-infrared spectroscopy study. Respir Physiol Neurobiol.

[37] Liu S, Zhang R (2022). Aerobic exercise alleviates the impairment of cognitive control ability induced by sleep deprivation in college students: research based on Go/NoGo Task. Front Psychol.

[38] Lu L, Dong M, Jian SY, Gao J, Ye LZ, Chen HR (2021). Sex differences in the factors associated with sleep duration in university students: a cross-sectional study. J Affect Disord.

[39] Mato M, Tsukasaki K (2020). Relationship between breakfast consumption and health-related habits among university students in Japan. Jpn J Public Health.

[40] Pengpid S, Peltzer K (2019). Prevalence and associated factors of skipping breakfast among university students from 28 countries: a cross-sectional study. Int J Adolesc Med Health.

[41] Haddad M, Menhas R (2025). Physical activity as a mediator between sleep, mental health, and well-being in active individuals. Physical activity and sports as preventive medicine for psychosocial health and well-being.

[42] Wang X, Lei SM, Le S, Yang Y, Zhang B, Yao W (2020). Bidirectional influence of the COVID-19 pandemic lockdowns on health behaviors and quality of life among Chinese adults. Int J Environ Res Public Health.

[43] Zhang Y, Liu J, Zhang Y, Ke L, Liu R (2022). Interactive compensation effects of physical activity and sleep on mental health: a longitudinal panel study among Chinese college students during the COVID-19 pandemic. Int J Environ Res Public Health.

[44] Zhang B, Lei SM, Le S, Gong Q, Cheng S, Wang X (2022). Changes in health behaviors and conditions during COVID-19 pandemic strict campus lockdown among Chinese university students. Front Psychol.

[45] Hammoudi SF, Mreydem HW, Ali BTA, Saleh NO, Chung S, Hallit S (2021). Smartphone screen time among university students in Lebanon and its association with insomnia, bedtime procrastination, and body mass index during the COVID-19 pandemic: a cross-sectional study. Psychiatry Investig.

[46] Iqbal S, Alanazi RF, Alahmed AH, Alnakhli AF, Alghanim MH, Alghamdi MA (2021). Prevalence of sleep disturbance and anxiety due to the COVID-19 pandemic in Saudi Arabia. Sleep Sci.

[47] Xiao P, Chen L, Dong X, Zhao Z, Yu J, Wang D (2022). Anxiety, depression, and satisfaction with life among college students in China: nine months after initiation of the outbreak of COVID-19. Front Psychiatry.

[48] Ren Z, Xin Y, Ge J, Zhao Z, Liu D, Ho RCM (2021). Psychological impact of COVID-19 on college students after school reopening: a cross-sectional study based on machine learning. Front Psychol.

[49] Hosen I, Mamun F, Mamun MA (2021). The role of sociodemographics, behavioral factors, and internet use behaviors in students’ psychological health amid COVID-19 pandemic in Bangladesh. Health Sci Rep.

[50] Su J, Wei E, Clark C, Liang K, Sun X (2022). Physical exercise, sedentary behaviour, sleep and depression symptoms in Chinese young adults during the COVID-19 pandemic: a compositional isotemporal analysis. Int J Ment Health Promot.

[51] Nakahara-Gondoh Y, Tsunoda K, Fujimoto T, Ikeda T (2022). Effect of encouraging greater physical activity on number of steps and psychological well-being of university freshmen during the first COVID-19-related emergency in Japan. J Phys Educ Sport.

[52] Al-Musharaf S, Aljuraiban G, Bogis R, Alnafisah R, Aldhwayan M, Tahrani A (2021). Lifestyle changes associated with COVID-19 quarantine among young Saudi women: a prospective study. PLoS One.

[53] Rafraf M, Molani-Gol R, Sahebjam M (2023). Effect of COVID-19 pandemic on eating habits and lifestyle of college students in Tabriz, Iran: a cross-sectional study. Front Public Health.

[54] Tian V, Ramlee F, Hamzah H (2025). Relationship between physical activity, mood and sleep quality: ecological momentary assessment study among young adults. Int J Dev Sci.

[55] Pankowska MM, Lu H, Wheaton AG, Liu Y, Lee B, Greenlund KJ (2023). Prevalence and geographic patterns of self-reported short sleep duration among US adults, 2020. Prev Chronic Dis.

[56] Australian Institute of Health and Welfare (2021). Sleep problems as a risk factor for chronic conditions.

[57] Zhang Y, Zhang H, Ma X, Di Q (2020). Mental health problems during the COVID-19 pandemics and the mitigation effects of exercise: a longitudinal study of college students in China. Int J Environ Res Public Health.

[58] Qaiser S, Daud MNM, Ibrahim MY, Gan SH, Rahman MS, Sani MHM (2020). Prevalence and risk factors of prehypertension in university students in Sabah, Borneo Island of East Malaysia. Medicine.

[59] Wang L, Li N, Heizhati M, Li M, Pan F, Yang Z (2021). Prevalence and predictive nomogram of depression among hypertensive patients in primary care: a cross-sectional study in less developed Northwest China. Medicine.

[60] Maestri M, Romigi A, Schirru A, Fabbrini M, Gori S, Bonuccelli U (2019). Excessive daytime sleepiness and fatigue in neurological disorders. Sleep Breath.

[61] Lee DW, Jang TW, Kim HR, Kang MY (2021). The relationship between working hours and lifestyle behaviors: evidence from a population-based panel study in Korea. J Occup Health.

[62] Schumacher M, Sipes D (2015). The effects of sleep deprivation on memory, problem solving and critical thinking: an ex-post facto experimental study.

[63] Wise MJ (2018). Naps and sleep deprivation: why academic libraries should consider adding nap stations to their services for students. New Rev Acad Librariansh.

[64] Albqoor MA, Shaheen AM (2021). Sleep quality, sleep latency, and sleep duration: a national comparative study of university students in Jordan. Sleep Breath.

[65] Sook Huey L, Ramlee F, Othman A (2025). Battling sleep disturbances and academic procrastination in undergraduates: a pilot study on acceptance and commitment therapy vs. motivational interviewing. Behav Sleep Med.

[66] Ghrouz AK, Noohu MM, Manzar MD, Spence DW, BaHammam AS, Pandi-Perumal SR (2019). Physical activity and sleep quality in relation to mental health among college students. Sleep Breath.

[67] Pengpid S, Peltzer K (2020). Vigorous physical activity, perceived stress, sleep and mental health among university students from 23 low- and middle-income countries. Int J Adolesc Med Health.

[68] Balhareth A, Jafer M, van der Borgh-Sleddens E, Kremers S, Meertens R (2021). Determinants of weight-related behaviors in male Saudi university students: a qualitative approach using focus group discussions. Int J Environ Res Public Health.

[69] Bisson ANS, Lachman ME (2023). The relationship of daily physical activity and sleep in adults: variations by age, sex, and race. J Behav Med.

[70] Wang R, He L, Xue B, Wang X, Ye S (2022). Sleep quality of college students during COVID-19 outbreak in China: A cross-sectional study. Altern Ther Health Med.

[71] Tang NKY, McEnery KAM, Chandler L, Toro C, Walasek L, Friend H (2022). Pandemic and student mental health: mental health symptoms among university students and young adults after the first cycle of lockdown in the UK. BJPsych Open.

[72] Yang GY, Lin XL, Fang AP, Zhu HL (2021). Eating habits and lifestyles during the initial stage of the COVID-19 lockdown in China: a cross-sectional study. Nutrients.

[73] Zheng C, Huang WY, Sheridan S, Sit CHP, Chen XK, Wong SHS (2020). COVID-19 pandemic brings a sedentary lifestyle in young adults: a cross-sectional and longitudinal study. Int J Environ Res Public Health.

[74] Abouzid M, El-Sherif DM, Eltewacy NK, Dahman NBH, Okasha SA, Ghozy S (2021). Influence of COVID-19 on lifestyle behaviors in the Middle East and North Africa Region: a survey of 5896 individuals. J Transl Med.

[75] Salman A, Sigodo KO, Al-Ghadban F, Al-Lahou B, Alnashmi M, Hermassi S (2021). Effects of COVID-19 lockdown on physical activity and dietary behaviors in Kuwait: a cross-sectional study. Nutrients.

[76] Dai W, Zhou J, Li G, Zhang B, Ma N (2021). Maintaining normal sleep patterns, lifestyles and emotion during the COVID-19 pandemic: The stabilizing effect of daytime napping. J Sleep Res.

